# Unveiling Frequently Co-Occurring Reasons of Attitudinal Acceptance of Intimate Partner Violence against Women: A Behavioral Data Science Perspective

**DOI:** 10.3390/ijerph191912429

**Published:** 2022-09-29

**Authors:** Muhammad Yasir, Ayesha Ashraf, Muhammad Umar Chaudhry, Syeda Azra Batool, Syeda Shahida Batool, Elzbieta Jasinska, Zbigniew Leonowicz, Michal Jasinski

**Affiliations:** 1 Department of Computer Science, Faisalabad Campus, University of Engineering and Technology Lahore, Faisalabad 38000, Pakistan; 2 Department of Economics, The Women University, Multan 60000, Pakistan; 3 Department of Computer Science, MNS-University of Agriculture, Multan 60000, Pakistan; 4 School of Economics, Bahauddin Zakariya University, Multan 60000, Pakistan; 5 Department of Psychology, GC University, Lahore 54000, Pakistan; 6 Department of Operations Research and Business Intelligence, Wroclaw University of Science and Technology, 50-370 Wroclaw, Poland; 7 Department of Electrical Engineering Fundamentals, Faculty of Electrical Engineering, Wroclaw University of Science and Technology, 50-370 Wroclaw, Poland

**Keywords:** intimate partner violence (IPV), gender-based violence, victimization, behavioural data science (BDS), frequent itemset mining, data mining

## Abstract

The results of gender equality indicators across the world in the form of prevalence of intimate partner violence (IPV) against women are striking and has thus drawn the attention of policy makers as well as necessitates the adoption of a comprehensive system to deal with. The situation of IPV in Pakistan is alarming. This study examines the acceptability attitude of women and men toward intimate partner violence against women through data science. It discovers and contrasts the frequently co-occurring reasons due to which husbands’ behaviour of beating their wives is believed to be legitimate by both partners in the province of Punjab, Pakistan. Though the discovered frequently co-occurring reasons, such as “arguing with the husband and neglecting the children” altogether, are similar in both genders but the fraction of wives believing in such reasons are significantly greater than that of husbands. This psychological disparity across genders could help in identifying the social and cultural factors to whom this disparity is attributed. It is expected that the identified co-occurring groups of reasons would help to understand the problem to the next level and devise better strategies to mitigate them.

## 1. Introduction

According to United Nations (UN), one in every five females aged between 15 and 49 reports a case of intimate partner violence (IPV), physical or sexual, within a 12-month period. As per a WHO multicountry study on women’s health and domestic violence against women, the rate of women experienced physical and/or sexual violence by an intimate partner at some point in their lives ranged between 15% and 71% around the globe [1]. Attitudinal acceptance of IPV exacerbates the existing gender inequalities by increasing the prevalence of IPV. Studies have shown that attitudes toward IPV against women is one of the most prominent predictors of the prevalence of IPV [2,3,4]. It is suggested that behavioural transformation can play an important role in eradication of this serious issue [5,6]. A deep understanding of acceptance of this violent behaviour therefore provides a pathway to achieve the sustainable development goal related to gender equality.

The justifications for attitudinal acceptance of intimate partner violence against women vary among different societies, both developed and developing [7,8]. It is argued that social norms and beliefs about traditional gender roles shape attitudes towards IPV acceptance. Transgressions are the triggers for women to blame themselves for violence and for men to provide justification to discipline their wives [9]. In addition, research has proposed that schooling [10,11], gender [12,13], age [3,14], and residence [15,16] may account for differences in attitude toward IPV. Factors such as education, equitable societal norms, discouragement of early marriages, among others are found to have a significant role in altering the behavior of women from acceptance to non-acceptance of violence.

The situation of IPV in Pakistan is alarming. According to the Pakistan Demographic and Health Survey (PDHS, 2017–2018), the prevalence of IPV of ever-partnered women and girls aged 15 years in the last 12 months is 24.8 percent. The survey report is publicly available at https://dhsprogram.com/pubs/pdf/FR354/FR354.pdf (accessed on 1 June 2022). The most common type of IPV women have ever experienced is psychological/emotional violence (20.6%), followed by physical violence (13.6%) and sexual violence (3.6%). Intimate partner violence has negative implications on survivor’s wellbeing, and it has been well noted, including the impacts on reproductive, physical, and mental health.

The women in Pakistan having experiences of IPV are more likely to have a miscarriage (6.7%), stillbirth or abortion (5.3%), higher acute illness and acute pain scores, suicidal tendencies (9.3%), and depression in comparison to women with similar characteristics but having no experience of IPV [17]. The physiological and health impacts of violence against women and girls (VAWG) also have economic and social costs that not only impact individual women and their families but also ripple through society and the economy at large. Women who experience one or more forms of VAWG miss more days of work and are less productive than women who do not suffer violence.

The average days lost due to absenteeism of women experiencing IPV are 31.67 days, whereas average days lost due to presentism of women experiencing IPV are 23.10 days. Women reported missing care work for approximately 11 million days due to domestic violence, including more than 6 million days due to IPV. One in seven female employees in the businesses surveyed reported productivity loss because of IPV—equal to 17 days per employee in the last year. The national loss in productivity due to VAWG in Pakistan was 80 million days annually, equivalent to 2.2% of employed women in effect not working and if only days of absenteeism are considered, households lost nearly US$146 m in income annually due to VAWG [17].

The perpetration of violence Influences men’s working patterns and productivity as well, suggesting that IPV has a cost for businesses with a male workforce. IPV also impacts children’s mental health and the education of the children of women experiencing violence. Nearly 24% reported that their children felt scared and 21% reported that their children felt confused. Smaller percentages also reported other symptoms, such as not wanting to play, wetting the bed, having nightmares, and physically shaking. Due to IPV, children missed nearly 2.5 million days in a year. Poor mental health and absenteeism from school has a long-term impact on the depth and quality of human capital of the next generation. Studies from different patriarchal societies have identified a common set of roles expected from women, such as proper cooking, childcare, seeking permission from husbands or other family members before going out, not arguing with husbands, and meeting the sexual need of husbands. If the wives do not meet the husbands’ expectations, they eventually become the reasons that lead to IPV against women consisting of physical torture by husbands predominantly.

Several techniques based on data science and machine learning have been employed in the recent past to address the IPV problem. Based on data mining, a comprehensive study has been done to reveal the relationship of IPV and Traumatic Brain Injury (TBI) using electronic health records (HER) data [18]. The author claims that HER data is more reliable as it comes from a third party, the clinicians, who do diagnoses and findings on patient visits. Results of the study suggest that health conditions due to to malnutrition, acquired thrombocytopenia, post-traumatic wound infection, local infection of wound, poisoning by cardiovascular drug, alcoholic cirrhosis, alcoholic fatty liver, and drug-induced cirrhosis were highly significant when IPV and TBI co-occurred.

Xue et al. [19] applied latent Dirichlet allocation (LDA), an unsupervised machine-learning method, to identify the conversations and discussions regarding domestic violence on Twitter. They took a dataset of tweets and employed LDA to identify latent topics related to domestic violence. CountVectorizer method has been used to convert Twitter messages into a document-term matrix so that words that appear more frequently in domestic violence, which are related to gender-based violence (GBV) could be collected. Results indicate that people have adequately recognized the term, domestic violence, due to Twitter, but social helping services are not utilizing Twitter to provide effective information to affected people.

Rodríguez-Rodríguez et al. [20] model and forecast GBV using historical data (open access) by deploying machine learning methods; the proposed technique was experimented in three different Spanish regions and showed effective results. A study has been conducted to investigate the discussions of domestic violence on social media [21]. Topic modeliing and sentimental analysis was used to extract different subjects of discussion and for classifying tweets as positive or negative. Data mining techniques, such as hierarchical Dirichlet process, latent semantic analysis, and latent Dirichlet allocation, were used to explore the spikes in topic discussion that directly correspond to real world events. The words analysis from the domain-specific embeddings helped in obtaining insights of abuse types and the conditions experienced by the victims.

Machine learning models have been trained with self-reported data on IPV from different questionnaires for predicting IPV from free text [22]. A tool, namely DetectIPV, has been developed by using Universal Sentence Encoder (USE) with SVN, random forest, Naïve ayes, and logistic regression for detecting IPV in free text. The tool could help in characterizing the predictability of multiple violence types, such as sexual abuse, physical abuse, or emotional abuse from free text. The tool can identify physical abuse with the highest accuracy followed by sexual abuse; however, emotional abuse was found to be the most difficult to be predicted. Authors claim that the tool can be utilized in multiple applications, such as identifying IPV in social media, electronic health records, court records, transcripts of interviews, and in large corpuses of text for research purposes.

For diagnosing crimes against women in India, machine learning algorithms, such as KNN, decision trees, and naive Bayes have been used [23]. Results of this study helped in identifying the most crime-ridden region in the country. Models for classification, such as random forests, support vector machines (SVM), multinomial logistic regression, and naïve BayesPaper were used for predicting IPV [24]. For balancing the dataset SMOTE and random sub-sampling techniques were used, decision trees have been applied for predicting domestic violence recidivism [25]. Authors claimed that a high accuracy of prediction can be achieved even using limited input features. Decision trees and naïve Bayes were found to be the most commonly used techniques to identify crimes against women [26].

IPV against women is surely an outcome of occurrence of certain reasons, where each reason has a unique significance. However, to the best of our knowledge, the following interesting questions are still unanswered.

- Are the reasons behind IPV against women, independent or is there some association among them? In other words, is it likely that two or more reasons frequently occur together?
- If there exist associations among reasons, is there any statistical measure to extract the frequent co-occurring groups of reasons? In other words, how can the frequently co-occurring groups of reasons could be figured out from all co-occurring groups of reasons?
- Are the extracted frequently co-occurring groups of reasons of same significance across genders?

Frequently co-occurring groups of reasons could help to understand the IPV problem even better. Thus, it is hypothesized that IPV could also be triggered due to the occurrence of more than one reason jointly rather than a single reason only. Moreover, significance of identified co-occurring reasons could vary between genders. A particular co-occurring group of reasons could be more significant from husbands’ perspectives and vice versa.

Data science is equipped with an advanced mining technique, called frequent itemset mining (FIM) or co-occurrence grouping that identifies the hidden associations among the items in a database [27]. A co-occurrence can be described as a rule: “If item A occurs then item B is likely to occur too” or “A implies B”. Therefore, FIM could help in identifying the frequently co-occurring reasons due to which husbands believe that they rightly beat their wives, and wives believe that their husbands are justified in beating them. For instance, a thorough FIM investigation on 100 responses containing one or more reasons, reveals that 75 responses contain reasons “A” and “B” jointly. It means that 75% respondents believe that husbands rightly beat their wives if both reasons happen together. Thus, the support or frequency of co-occurrence of “A” and “B” (fraction of responses containing both “A” and “B”) is 75%; hence, there exists a strong association between these reasons, whose joint effect on IPV cannot be overlooked. Numerous such associations of varying significance could be identified furnishing deeper insights.

Hence, the aim of this study is to examine the acceptability attitude of women and men toward IPV against women in Punjab, Pakistan through FIM. It will help in demystifying the IPV problem by extracting all co-occurring or joint reasons due to which husbands’ behavior of beating their wives is believed to be legitimate by both. To the best of our knowledge, despite tackling the IPV problem in numerous ways, no such investigation has been done so far. For this purpose, a recently proposed FIM algorithm, deferring the generation of power sets for discovering frequent itemsets from sparse big data (D-GENE) [28] is independently applied to the responses of both husbands and wives. D-GENE is chosen due to its superior runtime efficiency and the least memory consumption. Identified frequently co-occurring reasons are then contrasted to reveal the disparity of perceptions and thinking patterns of both genders.

The outcomes of this study reveal that a large fraction of wives and husbands believe that instead of a single reason, there are multiple reasons that when occurring jointly husbands rightly beat their wives. Moreover, a clear distinction between the significance of identified frequently co-occurring reasons has been found across genders. Thus, it is envisaged that applying this data science technique will give deeper insights that will help in understanding and contrasting the complex psychological patterns when IPV happens, the acceptability of cultural and social norms, and potential societal pitfalls from the perspective of both men and women. Moreover, identification of the results will help to take pre-emptive measures to thwart this behavioral dilemma.

The paper is organized in the following manner. The methodology is presented in Section 2. Experimental details are presented in Section 3. Results are discussed in Section 4. Section 5 is dedicated to the detailed discussion. The conclusion is presented in Section 6.

## 2. Methods

This section is dedicated to the methodology of mining the frequently co-occurring reasons that husbands feel a right to beat their wives from the perspectives of both. In the first place, details about participants’ datasets and procedures are explained. The FIM method is discussed afterward, followed by the measures taken to pre-process and transform the data.

### 2.1. Study Particpants and Procedures

The data is obtained from Multiple Indicators Cluster Survey (MICS) 2018 supported by UNICEF and is publicly available at https://mics.unicef.org/surveys (accessed on 15 January 2022). The total sample size was 53,840 households. Out of Six questionnaires that were used in the survey, two questionnaires have been used in the current study. One of the questionnaires was for individual females aged 15 to 49 and the other was for individual males aged 15–49. We have included the data of males and females that responded “yes” against any of the five conditions included in the questionnaires. The reasons mentioned in the questionnaire are mentioned in Table 1. Some samples of the collected data containing participants’ responses are presented in Table 2 in which the first four records show co-occurring reasons; however, the last record contains a single reason.

### 2.2. Frequent Itemset Mining (FIM)

It is assumed that *I* = {*i*_1_, *i*_2_, … *i_n_*} represents a set that contains all items existing in a transactional database; *T* denotes a transaction consisting of some items of *I*, such that *T* ⊆ *I* and having a distinctive identifier TID; and *D* denotes a database, which is a set of transactions = {*T*_1_, *T*_2_, …, *T_n_*}. *S* denotes an itemset where *S* ⊆ *I*. *S* is also called a k-itemset, where |*S*| = k. *T* contains *S* if and only if *S* ⊆ *T*; the *support* of *S*, denoted as sup(*S*), is the percentage of transactions in *D* in which *S* exists.

FIM or co-occurrence grouping is an unsupervised technique that performs exhaustive search through the data for combination of items who entail interesting statistics [27]. The interestingness of a frequent itemset or a co-occurrence group is based on a constraint, called minimum *support* threshold (*minsup*), which is a statistical measure that is used to verify co-occurrence grouping. *S* is regarded as a frequent itemset if and only if *minsup* ≤ sup(*S*). It means that, in IPV perspective, not all co-occurring reasons would be regarded as frequent; instead, some of them whose *support* surpasses *minsup* are regarded as frequent, hence interesting. The task of frequent itemset mining is to explore all frequent itemsets with their *supports*, for a database *D* and at a particular *minsup* value [29]. The results of FIM are the descriptions of items that occur jointly.

### 2.3. Data Collection

To conduct experiments, two datasets were used. One dataset contained the responses of husbands, containing 6243 records. The other dataset contained the responded questionnaire of wives, containing 18,906 records. The features of the datasets are presented in Table 3. Experiments were performed on both datasets at different *minsup* values to identify interesting frequently co-occurring reasons.

### 2.4. Data Transformation

Both datasets are transformed into a required format to apply the DGENE algorithm. As an input, D-GENE takes a transactional dataset to identify frequent itemsets, where each transaction contains one or more items. In this study, each record that contains a participant’s response, is regarded as a transaction in which each reason will be denoted as an item. Thus, it can be stated that, in Table 3, husbands’ and wives’ datasets contain 6243 and 18,906 transactions, respectively. Afterward, each reason (item) mentioned in the questionnaire is assigned an item symbol, which is a discrete number, as shown by Table 4. Therefore, each record corresponding to a participant’s response is transformed into a transaction containing one or more numbers. After assigning an item symbol to each reason, the transformed version of Table 2 is shown in Table 5.

## 3. Implementation

After data transformation, the D-GENE algorithm was applied to both datasets to find the frequently co-occurring reasons (itemsets) at different *minsup* values. D-GENE works in three phases, at any user-specified *minsup* value. For better illustration, the following section demonstrates the working of D-GENE at *minsup* 38% for the dataset of husbands. Results are presented in the next section.

### 3.1. D-GENE Phase 1

Total records of husbands’ data set are 6243; thus, 38% of 6243 = 2372. Itemsets having *support* or frequency of occurrence ≥2372 will be regarded as frequent itemsets. A frequent 1-itemset depict a frequent single reason due to which husbands and wives believe that the husbands are justified to beat their wives. To find frequently co-occurring itemsets, identification of frequent 1-itemsets is essential. In the first phase, when D-GENE reads a transaction, it performs the below mentioned tasks to extract frequent 1-itemsets (itemsets containing a single item).

1. D-GENE compresses the dataset by keeping similar (repeated) transactions once. D-GENE reads each transaction and counts its *support*, then places the transaction and its *support* count in a dictionary abstract data type (ADT), named as D1, as a key value pair. A transaction usually occurs several times in a dataset, but D-GENE stores it as a key only once; however, its *support* count is increased by one on each occurrence. The data compression in this phase prevents the algorithm to perform the same processing repeatedly on each occurrence of a certain transaction in later phases; thus, it saves precious processing time and memory;
2. After placing a transaction (key) in D1, the *support* count of every single item of that transaction is calculated. D-GENE uses another dictionary ADT, D2, in which every single item is stored separately as a key whose value is set to 1 on its first occurrence. On subsequent occurrences of the same single item, its value is increased by one accordingly.

D-GENE repeats the above steps for each transaction. Finally, D-GENE filters the individual items whose *support* count or frequency of occurrence is ≥2372. Extracted frequent 1-itemsets are stored in S1, a set ADT. S1 is used in later phases of D-GENE to identify frequent co-occurring itemsets.

### 3.2. D-GENE Phase 2

During this phase, the algorithm reads a single key from D1 at a time, perform its intersection with S1, and stores the intersection as a key in another dictionary ADT namely, D3, with the value equal to value of the key read from D1. Intersection of each key with S1 trims the key by neglecting the infrequent items from it. The process is repeated for each key of D1. Thus, each transaction becomes a trimmed transaction containing only the frequent items and stored in D3.

### 3.3. D-GENE Phase 3

In the last phase, D-GENE reads one trimmed transaction (key) from D3 at a time, makes its ITTL by generating its power set, and stores each subset into a dictionary ADT namely, D4, as a key with the value equal to the value of the currently read key from D3. Afterward, the value of the subset is compared with the *minsup*. If the value of the subset is greater than or equal to *minsup*, it is regarded as a frequent itemset, deleted from D4, and stored in F, which is a set ADT to store all frequent itemsets. Once a subset is stored in F, it cannot be stored again in D4. However, during any subsequent ITTL generation, if a subset is not frequent, its existing value is incremented by the key value read at that moment of time from D3. D-GENE repeats the process for each key of D3. Finally, F is printed describing all frequent itemsets.

## 4. Results

Table 6 shows the output of the step 1 of phase 1 when *minsup* was set to 38% for husbands’ dataset. The transaction {1} occurs 456 times in the dataset, which means that according to 456 husbands, a husband is justified in beating his wife “If she goes out without telling him”. Thus, the *support* count of {1} is 456. Similarly, transaction {1,5} occurs 24 times, which means 24 husbands responded in the questionnaire that a husband rightly beats his wife “if she goes out without telling him and if she burns the food” jointly. Table 7 shows the output of step 2 of phase 1 in which each item of a transaction, which is just stored in D1 in phase 1 (shown by Table 6) is stored separately in D2 as a key with the value of that transaction.

After repeating the above steps for all keys in D1, the cumulative value of each item is calculated that shows its cumulative *support* count. For instance, item {1} appears alone in 456 transactions (row 1 in Table 6 and Table 7), and then it co-occurs with items {2}, {3}, {4}, and {5} in the transactions {1,2}, {1,3}, {1,4}, and {1,5}, having *support* counts of 315, 239, 68, and 24, respectively, as shown by rows 6–9 in Table 6 and Table 7. Later, it is found that item {1} has been co-occurred with other items in larger transactions present in rows 16–21, 26–29, and 31 respectively. Thus, the cumulative *support* count of item {1} can be calculated by adding the values of all keys in which {1} is present, whether alone or co-occurring with other items. Cumulative *support* counts of other items can also be calculated in the same manner. Table 7 shows that cumulative *support* counts of {1}, {2}, {3}, {4}, and {5} were 3900, 3946, 4425, 2889, and 2005, respectively. Afterwards, items {1},{2},{3}, and {4} are selected as frequent 1-itemsets because their cumulative *support* count is ≥2372, as shown in the last row of Table 7. However, itemset {5} is not selected because its cumulative *support* count is less than 2373.

Eradication of itemset {5} in this initial step shows that one of the reasons mentioned in the questionnaire, “if she burns the food”, was not believed to be a strong reason due to which husbands would beat their wives. Thus, according to antimonotone property [30], itemset {5} cannot be a frequent itemset at *minsup* 38%. Antimonotone property says that superset of an infrequent itemset cannot be a frequent itemset. Thus, co-occurring itemsets containing {5} cannot be frequent in later stage of the algorithm. Extracted frequent 1-itemsets are stored in S1, a set ADT, as shown in Table 8.

Output of the Phase 2 is presented in Table 8. The intersection of each transaction of Table 6 with S1 eradicates its infrequent part; thus, a trimmed transaction is saved in D3 present in Table 8. In case of re-occurrence of the same trimmed transaction (identical transaction), its value in D3 is incremented by its value read from D1 from Table 6. For instance, the first key in Table 8 is {1}, which is present at first row in Table 6, and the second key is {1,5}, which is present at row 9 in Table 6. Intersection of {1} with S1 returns {1} but intersection of {1,5} with S1 also returns {1}, which is a trimmed transaction due to the removal of item {5}. Both intersections result into identical transactions. Thus, D3 stores the identical transaction {1} as a key with a value by adding the values of {1} and {1,5}. Hence, the cumulative *support* count of itemset {1} is 480. The same procedure is repeated for subsequent identical trimmed transactions, as shown by Table 8.

Table 9 shows the output of the phase 3 of D-GENE algorithm that takes each itemset from D3, generates its ITTL showing the powerset, and stores each subset as a key in D4. In Table 9, each column under D4 represents a subset (key), where each key is now regarded as an itemset itself. Moreover, value of each key is equal to that of its itemset taken from D3. For instance, in Table 9, itemset {1,2} in D4 is a subset of powersets of {1,2}, {1,2,3} {1,2,4}, and {1,2,3,4} having values of 360, 733, 96, and 1732, respectively. After storing {1,2} in D4, as a subset of powersets of {1,2}, {1,2,3}, {1,2,4}, and {1,2,3,4}, its cumulative *support* count becomes 2921 exceeding the *minsup*; thus, {1,2} appeared as frequent, derived from D4, and saved in F. Later, the subset {1,2} will not be pushed into D4 again as it has already been declared frequent. Similarly, itemset {1,4} in D4 is the subset of powersets of {1,4}, {1,2,4}, {1,3,4}, and {1,2,3,4}. However, its cumulative *support* count is 2048, which is less than *minsup*. Therefore, itemset {1,4} is not a frequent itemset. After reading each key of D2, frequent itemsets are stored in F. Finally, the algorithm stops, and F is printed describing the frequent co-occurring itemsets.

Thus, according to Table 9, that frequent co-occurring itemsets are {1,2}, {1,3}, {2,3}, {3,4}, and {1,2,3} because their cumulative (total) *support* is greater than *minsup*. Hence, it was concluded that 38% of husbands believe that they are justified in beating their wives due to the following frequently co-occurring reasons.

1. {1,2}⇒ If she goes without telling him *and* if she neglects the children;
2. {1,3}⇒ If she goes out without telling him *and* if she argues with him;
3. {2,3}⇒ If she neglects the children *and* if she argues with him;
4. {3,4}⇒ If she argues with him *and* if she refuses to have sex with him;
5. {1,2,3}⇒ If she goes without telling him *and* if she neglects the children *and* if she argues with him.

D-GENE was run several times on husbands’ dataset to observe the frequently co-occurring reasons at different *minsup* values, as shown in Table 10. The most frequent 1-itemset is {3}, which means that 70% husbands believe that a husband is justified to beat his wife “if she argues with him”. The most frequently co-occurring itemset is {2,3}, which means that 48% husbands believe that a husband is justified to beat his wife “if she neglects the children *and* if she argues with him” altogether. It can be inferred from Table 10 that out of 70% transactions in which itemset {3} is present, there exist 48% of transactions in which itemset {2} is co-occurring; thus, making itemset {2,3} a frequently co-occurring itemset.

As *minsup* tends to decrease, more interesting co-occurring situations arise, such as 46% of husbands believe that they rightly hit or beat their wives:

1. {1,2} => If she goes out without telling him *and* if she neglects the children;
2. {1,3} => If she goes out without telling him *and* if she argues with him;
3. {2,3} => If she neglects the children *and* if she argues with him.

Similarly, D-GENE has been run several times on wives’ data set to get the frequently co-occurring reasons from wives’ perspectives at different *minsup* thresholds. The most frequent 1-itemset is {3}, which means that 76% of wives believe that a husband is justified in beating his wife “if she argues with him”. The most frequent co-occurring itemset is {2,3}, which means that 60% of wives believe that a husband is justified to hit or beat his wife “if she neglects the children and argues with him” altogether. As *minsup* tends to decrease, more interesting co-occurring situations arise, such as 55% wives believe that they are rightly hit or beaten by their husbands:

1. {1,2} => If she goes out without telling him *and* if she neglects the children;
2. {2,3} => If she neglects the children *and* if she argues with him;
3. {3,4} => If she argues with him *and* if she refuses to have sex with him.

Table 10 and Table 11 reveal the following disparities between perceptions across genders based on frequently co-occurring reasons.

1. In Table 10, from husbands’ perspective, co-occurrence grouping starts at *minsup* 48%. It means that 48% of husbands believe that a single condition is not sufficient to justify that they rightly beat their wives;
2. In Table 11, from wives’ perspectives, co-occurrence grouping starts at *minsup* 60%. It means that more than 50% of wives believe that a single reason is not sufficient for their husbands to justify that they rightly beat their wives;
3. The most frequently co-occurring itemset in both tables is {2,3}, which means that:
  - A husband believes that he is justified to beat his wife “if she neglects the children *and* if she argues with him” jointly;
  - A wife also believes that her husband is justified in beating her “if she neglects the children *and* If she argues with him” jointly.

Though the results coincide but the difference in fraction of both genders who believe in this co-occurring situation is significant, e.g., husbands are 48%, while wives are 60%. It means that more than 50% of wives believe that a single reason (whether she neglects the children or if she argues with him) is not suffice for their punishment by their husbands, but only 48% husbands think alike.

4. Another frequently co-occurring situation belongs to the itemset {3,4}, which means that:
  - A husband believes that he is justified to beat his wife “if she argues with him *and* if she refuses to have sex with him” jointly;
  - A wife also believes that her husband is justified in beating her “if she argues with him *and* if she refuses to have sex with him” jointly.

The key thing is the fraction of husbands and wives who have chosen the itemset {3,4}. From Table 10, it is evident that the husbands are 38% who have chosen the itemset {3,4}; however, itemset {3,4} looks a lot more frequent (55%) in Table 11 that belongs to wives’ dataset. This huge difference between the fraction of husbands and wives who believe in this co-occurring situation is significant. It can be concluded that, “55% wives believe that a single reason (if she argues with him or if she refuses to have sex with him) is not sufficient for the husbands to justify that they rightly beat their wives. On the contrary, only 38% of husbands believe alike, which means that according to more than 50% of husbands, a single reason (if she argues with him or if she refuses to have sex with him) is sufficient to beat their wives. Thus, a huge disparity is found for the co-occurring itemset {3,4}.

5. Furthermore, a frequent co-occurring situation belonging to the itemset {2,4} is also important, which states that:
  - A husband believes that he is justified to beat his wife “if she neglects the children *and* if she refuses to have sex with him.”
  - A wife also believes that her husband is justified in beating her “if she neglects the children *and* if she refuses to have sex with him.”

The fraction of husbands and wives who have chosen the itemset {2,4} is interesting. From Table 10, it is evident that only 34% of husbands believe that they are justified in beating their wives “if they neglect the children *and* refuse to have sex with them” altogether. However, the itemset {2,4} looks a lot more frequent (51%) in Table 11 that belongs to wives.

## 5. Discussion

Concerning the frequently co-occurring reasons of IPV against women in Pakistan, both wives and husbands sampled believed that there was more than one reason for woman battering. Approximately 50% of men and more than 50% of women believed that a single condition was not sufficient to justify that they rightly beat their wives (see Table 10 and Table 11). This tendency of co-occurrence of two or more than two reasons of women battering indicates that both genders believe that husbands’ behaviors and women’s passive attitudes toward violence is due to their strong belief in the justified reasons of battering.

The most frequently co-occurring itemset appeared in Table 10 and Table 11, which was {2,3}, meaning that both wives and husbands believe that wife battering is justified if the wife neglects the children *and* if she argues with the husband. It reflects the central place of children in the marital relationship in Pakistan and it also replicates that in Pakistani society children determine the marital quality of their parents. At the same time, wives are expected to be modest in Pakistani culture. Right from childhood, it is inculcated in the minds of girls that they should not speak loudly and should remain modest, and boys are encouraged to raise their voices for their rights and they are treated as superior to girls.

The second frequently co-occurring situation belongs to the itemset {3,4}, which means that wives and husbands both believe that a husband is justified to beat his wife if she argues with him *and* if she refuses to have sex with him” It again reflects the supremacy of the husband in the marital relationship. In a patriarchal society, such as Pakistan, power in the marital relationship lies with the husband. He wants to shape the behavior and attitude of his wife according to his own will and does not give her a right to give her view point in day-to-day matters and difference of opinion is discouraged. However, only 34% of husbands’ in the sample have gone with dual reasons as justification to beat wives; it means that a majority of husbands believe that they do not need to have multiple reasons to beat their wives, rather a single reason could produce justification to beat women.

Furthermore, the third frequently co-occurring situation belonging to the itemset {2,4} is also important, which reflects that both partners believe that a husband is justified to beat his wife “if she neglects the children *and* if she refuses to have sex with him”. Here again women outnumbered men, which shows the conviction of men that a single reason can validate women battering. It reflects that husbands lose their tempers if kids are mishandled by wives and they do not take care of them and when they are inclined towards sex but their wives refuse. It shows the centrality of a sexual relationship in marriages for husbands.

The disparity in perception of both genders can be attributed to multiple factors, such as lack of communication and societal and cultural norms. Mutual communication between a couple is essential for mutual understanding, healthy marital relationship, and well-being. The occurrence of IPV is higher in the case of low and very low family communication and satisfaction. Violence can incite emotional distance, and this emotional detachment and lack of intimacy and communication may further lead to violence, so there is a vicious circle [31]. Communication leads to marital satisfaction [32] and if married partners are satisfied in their relationship, they will not abuse each other. Studies have shown that besides income and education, marital satisfaction is primarily the ability of couples to communicate [33].

Disparity in the perception of reasons for violence may also be attributed to the indigenous cultural norms of Pakistan, where male partners mostly dominate and do not allow their wives to raise their voice for their rights and give opinions in household decision making. The culture of patriarchy or the ideology regarding male superiority may manifest in different gender roles and hierarchy for example, aggression, male sexual entitlement, women battering as a right of a man, and the low social status and control of women. These expressions may lead to the process of enforcement of men as superior in the hierarchy and implementation of conservative female gender roles and their lower rank, as well as to the crisis of relationship conflict [34].

The analysis confirms that the reasons behind IPV against women are not independent; instead, there exist strong associations among them. Results are evident that often IPV against women happens due to the joint occurrence of multiple reasons. Moreover, a clear distinction in the significance of extracted co-occurring reasons has been observed across genders. It is revealed that various co-occurring reasons that were believed to be extremely significant by wives were not as significant from husbands’ perspectives. Thus, a clear disparity of perceptions and behaviors is found across intimate partners. The results exhibit a clear manifestation of hypothesis and open new avenues of analyzing the IPV problem.

This study has addressed IPV issue in a novel manner; however, it lacks analysis based on different age groups and living conditions, such as urban and rural and partners’ economic conditions. Analyzing the data based on these factors would bring in even deeper insights. Inclusion of these factors while discovering co-occurring reasons could be an interesting question for future research.

### Implications

The study has implications for marital counsellors to monitor the co-occurring reasons of intimate partner violence against women, while devising counselling plans. Disparity in giving the justification of IPV indicates that most of the time aggressive behaviors in marital relationships occur due to a lack of communication, so awareness regarding the significance of healthy communication in healthy and happy marriages should be spread. Results indicate that both married partners believe that IPV is not occurring instinctively, but there are some grounds for this battering behaviour, so there is a need to either mitigate the intensity of perceived reasons that justify IPV or women should modify their behaviour positively if they want to stop these aggressive acts of their husbands.

## 6. Conclusions

The situation of Intimate Partner Violence (IPV) in Pakistan is becoming worse. Attitudinal acceptance of IPV further deteriorates the situation because it brings a plethora of problems for the whole society. Among the most common facets of IPV against women in Pakistan, physical violence is in the second position, following psychological/emotional violence, and is triggered due to the occurrence of different reasons. As of today, these reasons were treated independently from each other; therefore, the questions such as, do husbands beat their wives due to multiple reasons frequently occurring together, and how significant are these co-occurring reasons according to husbands and wives, are still unanswered. The combined or co-occurring effect of reasons reveals the associations or relationships among reasons and thus cannot be ruled out. Based on frequent itemset mining (FIM), this study is a novel attempt to figure out the hidden associations among different reasons due to which the beating behaviour of husbands is felt justified by both husbands and wives. Rigorous experimentation has been carried out to prove that besides single reasons, a large fraction of wives and husbands believe that husbands’ beating behavior is triggered when multiple reasons occur together. Moreover, varying significance of each frequently co-occurring group of reasons has been identified across intimate partners by contrasting the results at different threshold values. Results show a significant disparity between perceptions of both genders that is attributed to numerous societal, cultural, and psychological factors. It is envisaged that the identification of co-occurrence grouping of these reasons would help in understanding the problem to the next level. IPV against women is severely harming public health. It affects women’ health in various ways whose consequences are born by the whole family, such as children’s mental health and education, and loss of productivity leading to negative economic conditions. The results reveal interesting eye-opening insights that could help practitioners and policy makers to devise new avenues of mitigating IPV and taking precautionary measures.

## Figures and Tables

**Table 1:** Conditions stated in the survey.

No.	Reasons
1	If she goes out without telling him
2	If she neglects the children
3	If she argues with him
4	If she refuses to have sex with him
5	If she burns the food

**Table 2:** Sample Responses of participants.

Participant No.	Questionnaire Response
1	If she goes out without telling him *and* If she argues with him
2	If she argues with him *and* If she burns the food
3	If she neglects the children *and* If she argues with him *and* if she burns the food
4	If she goes out without telling him *and* If she refuses to have sex with him
5	If she argues with him

**Table 3:** Dataset details.

No.	Belongs to	No. of Records
1	Husbands	6243
2	Wives	18,906

**Table 4:** Item symbols attached to the reasons.

Sr. No.	Item Symbol	Reasons
1	1	If she goes out without telling him
2	2	If she neglects the children
3	3	If she argues with him
4	4	If she refuses to have sex with him
5	5	If she burns the food

**Table 5:** The data transformation of Table 2.

Participant No.	Responded Questionnaire
1	1, 3
2	3, 5
3	2, 3, 5
4	1, 4
5	3

**Table 6:** Husbands’ dataset compression: storing similar transactions once in D1.

No.	Transaction (Key)	*Support* Count (Value)
1	{1}	456
2	{2}	302
3	{3}	640
4	{4}	179
5	{5}	128
6	{1,2}	315
7	{1,3}	239
8	{1,4}	68
9	{1,5}	24
10	{2,3}	282
11	{2,4}	95
12	{2,5}	44
13	{3,4}	225
14	{3,5}	59
15	{4,5}	36
16	{1,2,3}	586
17	{1,2,4}	73
18	{1,2,5}	45
19	{1,3,4}	104
20	{1,3,5}	40
21	{1,4,5}	13
22	{2,3,4}	156
23	{2,3,5}	47
24	{2,4,5}	17
25	{3,4,5}	51
26	{1,2,3,4}	515
27	{1,2,3,5}	147
28	{1,2,4,5}	23
29	{1,3,4,5}	35
30	{2,3,4,5}	82
31	{1,2,3,4,5}	1217
Total		6243

**Table 7:** Husbands’ data set: Cumulative *support* count of each item in every transaction and storing in D2.

No.	Itemset (Key)		*Support* Count of Each Item of a Transaction (Value)									
1	{1}	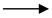	456									
2	{2}	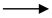	302									
3	{3}	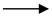	640									
4	{4}	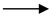	179									
5	{5}	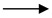	128									
6	{1,2}	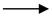	{1}	315		{2}	315					
7	{1,3}	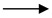	{1}	239		{3}	239					
8	{1,4}	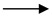	{1}	68		{4}	68					
9	{1,5}	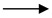	{1}	24		{5}	24					
10	{2,3}	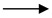	{2}	282		{3}	282					
11	{2,4}	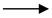	{2}	95		{4}	95					
12	{2,5}	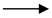	{2}	44		{5}	44					
13	{3,4}	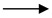	{3}	225		{4}	225					
14	{3,5}	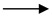	{3}	59		{5}	59					
15	{4,5}	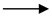	{4}	36		{5}	36					
16	{1,2,3}	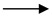	{1}	586	{2}	586	{3}	586				
17	{1,2,4}	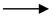	{1}	73	{2}	73	{4}	73				
18	{1,2,5}	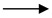	{1}	45	{2}	45	{5}	45				
19	{1,3,4}	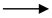	{1}	104	{3}	104	{4}	104				
20	{1,3,5}	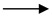	{1}	40	{3}	40	{5}	40				
21	{1,4,5}	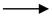	{1}	13	{4}	13	{5}	13				
22	{2,3,4}	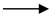	{2}	156	{3}	156	{4}	156				
23	{2,3,5}	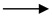	{2}	47	{3}	47	{5}	47				
24	{2,4,5}	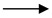	{2}	17	{4}	17	{5}	17				
25	{3,4,5}	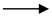	{3}	51	{4}	51	{5}	51				
26	{1,2,3,4}	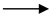	{1}	515	{2}	515	{3}	515		{4}		515
27	{1,2,3,5}	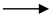	{1}	147	{2}	147	{3}	147		{5}		147
28	{1,2,4,5}	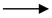	{1}	23	{2}	23	{4}	23		{5}		23
29	{1,3,4,5}	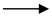	{1}	35	{3}	35	{4}	35		{5}		35
30	{2,3,4,5}	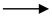	{2}	82	{3}	82	{4}	82		{5}		82
31	{1,2,3,4,5}	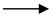	{1}	1217	{2}	1217	{3}	1217	{4}	1217	{5}	1217
Cumulative *support* count			{1}	3900	{2}	3946	{3}	4425	{4}	2889	{5}	2008
Frequent (Y/N)			Y		Y		Y		Y		N	

**Table 8:** Cumulative *support* of identical trimmed transactions of husbands dataset at *minsup* 38%.

D1			S1	Repeat the Following Steps for Each Key of D1. Take Intersection of Each Key of D1with S1. Store the Intersection Immediately in D3 as a Key along with the Its D1 Value.		D3 Each Key Is Stored in D3 only Once. Each Subsequent Arrival of a Key Results into the Addition of Its Value with the Value of the Same Key Stored already in D3. Thus, the Total Value of Key {1} in D3 Becomes 480. The Process Is Repeated for Every Intersection.	
No.	Key	Value		Key	Value = D1 (Value)	Key	Value
1	{1}	456	{1,2,3,4}	{1}	456	{1}	456 + 24 = 480
2	{1,5}	24	{1,2,3,4}	{1}	24		
3	{2}	302	{1,2,3,4}	{2}	302	{2}	302 + 44 = 346
4	{2,5}	44	{1,2,3,4}	{2}	44		
5	{3}	640	{1,2,3,4}	{3}	640	{3}	640 + 59 = 699
6	{3,5}	59	{1,2,3,4}	{3}	59		
7	{4}	179	{1,2,3,4}	{4}	179	{4}	179 + 36 = 215
8	{4,5}	36	{1,2,3,4}	{4}	36		
9	{1,2}	315	{1,2,3,4}	{1,2}	315	{1,2}	315 + 45 = 360
10	{1,2,5}	45	{1,2,3,4}	{1,2}	45		
11	{1,3}	239	{1,2,3,4}	{1,3}	239	{1,3}	239 + 40= 279
12	{1,3,5}	40	{1,2,3,4}	{1,3}	40		
13	{1,4}	68	{1,2,3,4}	{1,4}	68	{1,4}	68 + 13 = 81
14	{1,4,5}	13	{1,2,3,4}	{1,4}	13		
15	{2,3}	282	{1,2,3,4}	{2,3}	282	{2,3}	282 + 47 = 329
16	{2,3,5}	47	{1,2,3,4}	{2,3}	47		
17	{2,4}	95	{1,2,3,4}	{2,4}	95	{2,4}	95 + 17 = 112
18	{2,4,5}	17	{1,2,3,4}	{2,4}	17		
19	{3,4}	225	{1,2,3,4}	{3,4}	225	{3,4}	225 + 51 = 276
20	{3,4,5}	51	{1,2,3,4}	{3,4}	51		
21	{1,2,3}	586	{1,2,3,4}	{1,2,3}	586	{1,2,3}	586 + 147 = 733
22	{1,2,3,5}	147	{1,2,3,4}	{1,2,3}	147		
23	{1,2,4}	73	{1,2,3,4}	{1,2,4}	73	{1,2,4}	73 + 23 = 96
24	{1,2,4,5}	23	{1,2,3,4}	{1,2,4}	23		
25	{1,3,4}	104	{1,2,3,4}	{1,3,4}	104	{1,3,4}	104 + 35 = 139
26	{1,3,4,5}	35	{1,2,3,4}	{1,3,4}	35		
27	{2,3,4}	156	{1,2,3,4}	{2,3,4}	156	{2,3,4}	156 + 82 = 238
28	{2,3,4,5}	82	{1,2,3,4}	{2,3,4}	82		
29	{1,2,3,4}	515	{1,2,3,4}	{1,2,3,4}	515	{1,2,3,4}	515 + 1217 = 1732
30	{1,2,3,4,5}	1217	{1,2,3,4}	{1,2,3,4}	1217		
31	{5}	128	{1,2,3,4}	{ }	0		

**Table 9:** Generating powersets (ITTLs) in the last phase of D-GENE for the husbands dataset at *minsup* 38%.

D3			D4 (Total Records: 6243, *minsup* = 38% => 2372) Read Each key from D3, Make Its ITTL by Generating its Power Set. Store Each Subset into D4 Immediately as a Key with Value Equal to the Value of the Key in D3. If a Subset (key) Comes again, just Add Its Value into the Existing Value of that Subset in D4. Keys with the value ≥2372, will be Regarded as Frequent Itemsets.														
Key	Value		{1}	{2}	{3}	{4}	{1,2}	{1,3}	{1,4}	{2,3}	{2,4}	{3,4}	{1,2,3}	{1,2,4}	{1,3,4}	{2,3,4}	{1,2,3,4}
{1}	480	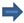	480														
{2}	346	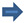		346													
{3}	699	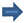			699												
{4}	215	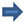				215											
{1,2}	360	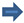	360	360			360										
{1,3}	279	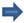	279		279			279									
{1,4}	81	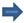	81			81			81								
{2,3}	329	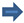		329	329					329							
{2,4}	112	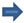		112		112					112						
{3,4}	276	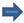			276	276						276					
{1,2,3}	733	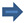	733	733	733		733	733		733			733				
{1,2,4}	96	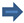	96	96		96	96		96		96			96			
{1,3,4}	139	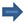	139		139	139		139	139			139			139		
{2,3,4}	238	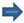		238	238	238				238	238	238				238	
{1,2,3,4}	1732	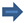	1732	1732	1732	1732	1732	1732	1732	1732	1732	1732	1732	1732	1732	1732	1732
Total support		Σ	3900	3946	4425	2889	2921	2883	2048	3032	2178	2385	2465	1828	1871	1970	1732
Frequent Itemsets			✓	✓	✓	✓	✓	✓	✕	✓	✕	✓	✓	✕	✕	✕	✕
			{1}	{2}	{3}	{4}	{1,2}	{1,3}		{2,3}		{3,4}	{1,2,3}				

**Table 10:** Frequently co-occurring reasons when husbands believe that they are justified in beating or hitting their wives based on husbands’ dataset.

minsup %	Frequent Itemsets
70	{3}
63	{2}, {3}
62	{1}, {2}, {3}
48	{1}, {2}, {3}, {2,3}
46	{1}, {2}, {3}, {4}, {1,2}, {1,3}, {2,3}
39	{1}, {2}, {3}, {4}, {1,2}, {1,3}, {2,3}, {1,2,3}
38	{1}, {2}, {3}, {4}, {1,2}, {1,3}, {2, 3}, {3,4}, {1,2,3}
34	{1}, {2}, {3}, {4}, {1,2}, {1,3}, {2, 3}, {2,4}, {3,4}, {1,2,3}
32	{1}, {2}, {3}, {4}, {5}, {1,2}, {1,3}, {1,4}, {2, 3}, {2,4}, {3,4}, {1,2,3}
31	{1}, {2}, {3}, {4}, {5}, {1,2}, {1,3}, {1,4}, {2, 3}, {2,4}, {3,4}, {1,2,3}, {2,3,4}
26	{1}, {2}, {3}, {4}, {5}, {1,2}, {1,3}, {1,4}, {2, 3}, {2,4}, {3,4}, {3,5}, {1,2,3}, {1,2,4}, {1,3,4}, {2,3,4}, {1,2,3,4}

**Table 11:** Frequently co-occurring situations when husbands believe that they are justified in beating or hitting their wives based on wives’ dataset.

minsup %	Frequent Itemsets
76	{3}
71	{2}, {3]
65	{1}, {2}, {3}
63	{1}, {2}, {3}, {4}
60	{1}, {2}, {3}, {4}, {2,3}
55	{1}, {2}, {3}, {4}, {1,2}, {2,3}, {3,4}
52	{1}, {2}, {3}, {4}, {1,2}, {1,3},{2,3},{3,4}
51	{1}, {2}, {3}, {4}, {1,2}, {1,3},{2,3}, {2,4}, {3,4}
50	{1}, {2}, {3}, {4}, {1,2}, {1,3},{2,3}, {2,4}, {3,4}, {1,2,3}
48	{1},{2}, {3}, {4}, {5}, {1,2}, {1,3},{2,3}, {2,4}, {3,4}, {1,2,3}, {2,3,4}
46	{1}, {2}, {3}, {4}, {5}, {1,2}, {1,3}, {1,4}, {2,3}, {2,4}, {3,4}, {1,2,3}, {2,3,4}
44	{1}, {2}, {3}, {4}, {5}, {1,2}, {1,3}, {1,4}, {2,3}, {2,4}, {3,4}, {3,5}, {1,2,3}, {1,3,4}, {2,3,4}
43	{1}, {2}, {3}, {4}, {5}, {1,2}, {1,3}, {1,4}, {2,3}, {2,4}, {2,5}, {3,4}, {3,5}, {1,2,3}, {1,2,4}, {1,3,4}, {2,3,4}

## Data Availability

Data publicly available at https://mics.unicef.org/surveys (accessed on 15 January 2022).
